# The effect of cigarette smoking and heated tobacco products on different denture materials; an in vitro study

**DOI:** 10.1186/s12903-025-05448-x

**Published:** 2025-02-02

**Authors:** Sara F. El Shafei, Ayman H. Amin, Eman G. Abdelghaffar, Sara Moataz, Fatma Makkeyah, Mohamed Shamel, Mahmoud Al Ankily

**Affiliations:** 1 Faculty of Dentistry, The British University in Egypt, Cairo, Egypt; 2 Fixed Prosthodontics Department, Faculty of Dentistry, The British University in Egypt, Cairo, Egypt; 3 Oral Biology Department, Faculty of Dentistry, The British University in Egypt, Cairo, Egypt

**Keywords:** Acrylic resins, Biofilm, Cigarette smoking, Denture bases, Tobacco products

## Abstract

**Purpose:**

This study compares the effect of conventional cigarette smoke versus heated tobacco on the discoloration, surface roughness, and bacterial colonization of different oral prosthesis materials.

**Materials and methods:**

A total of one hundred and twenty disc-shaped samples made of four different denture base materials were prepared to represent different denture bases to assess the surface roughness and biofilm formation; group (CA): conventional heat-cured acrylic resin (Acrostone, Egypt), group (FA): flexible acrylic resin (Valplast International Corp, USA), group (TA): heat-cured acrylic resin reinforced with titanium nanoparticles (TA nanoparticles, Nanogate, Egypt), and group (PA): 3D printed acrylic resin (Nexdent, The Netherlands). Another sixty samples of artificial and 3D printed teeth were used to assess the color change: conventional ready-made acrylic resin teeth (Acrostone, Egypt) and 3D-printed acrylic resin teeth (Nexdent, The Netherlands). Each group was further divided according to the smoking method into three subgroups (*n* = 10): the no-smoking exposure group (C), the conventional smoking exposure group (CS), and the heated tobacco exposure group (HT). A custom-made smoking device was used to perform the experiment. Six hundred cigarettes/heets representing 30 days of medium smoking behavior (20 cigarettes/day) were used. The surface roughness of the disc-shaped samples was measured before and after the experiment using the JITAI8101 surface roughness tester (Beijing Jitai Tech Detection Device Co., Ltd, China), and the color parameters were assessed before and after the experiment using VITA Easyshade Advance 4.01 (VITA shade, VITA made, VITA).

**Results:**

The results showed that both conventional cigarette smoking and heated tobacco increased the surface roughness of the denture base disc samples. This change was statistically significant in all sample groups. Bacterial accumulation was also increased in all four denture base sample groups, with the heated tobacco causing greater bacterial accumulation than conventional cigarette smoke. Regarding the color change, conventional smoking caused a more significant color change than heated tobacco for both types of teeth used.

**Conclusion:**

Both conventional smoke and heated tobacco affect dental materials adversely. Conventional cigarette smoking caused greater surface roughness and discoloration of the samples, while heated tobacco resulted in greater bacterial accumulation of study materials.

**Clinical implications:**

Increase dentists’ and patients’ awareness of the effects of different types of smoke.

## Introduction

In the oral environment, dental prostheses are continuously exposed to deleterious complex endogenous and exogenous factors that might result in biodegradation that alters the physical and mechanical properties of the material; one of these is cigarette smoking. According to the World Health Organization, cigarette smoking is a public health problem reported in almost 1.3 billion people around the world [1, 2], despite protracted anti-smoking campaigns, smoking remains an everyday habit.

Conventional cigarette smoke (CS) is composed of a mixture of a gaseous and a particulate phase and contains toxic agents such as carbon monoxide (CO) [3]. Pigments contained in tobacco residue (tar) can be responsible for the discoloration of both dental tissues and resin-based restorations [4–6]. Also, resin-based restorations may get contaminated by heavy metals such as lead and cadmium [7, 8] changing the chemical and physical properties such as surface roughness, water sorption, solubility, and staining [9–15].

Recently, new products known as “modified risk tobacco products” (MRTP) have been presented as an alternative to conventional cigarettes and an intermediate step in quitting the smoking habit, assuming that they contain a reduced number of harmful chemicals than regular CS [16], and many smoke users switched to these types of products. Therefore, the increasing use of MRTP leads to the need to evaluate the effects of such systems on the color stability of restoration materials and dental tissues [17].

Looking closely at both types of smoking to compare their effects, the smoke that directly emerges from a lit cigarette is frequently referred to as “whole smoke.” It comprises liquid droplets suspended in an aerosol mixture of gases and semi-volatile chemicals. This phase is called the particle phase. It is commonly known as “tar” or nicotine-free particulate fraction when it is devoid of nicotine. In comparison, E-cigarettes emit an aerosol that includes nicotine and other substances, but they don’t produce the same particle matter as conventional cigarettes. Consequently, these products are thought to stain less than conventional smoking [18–20].

Further comparisons between CS and HT show that CS results from incomplete tobacco combustion at temperatures reaching 900 °C. In contrast, the aerosols of heated tobacco are produced at temperatures well below 400 C. This significant difference in combustion temperatures alters the resulting chemical constituents produced, supposedly causing the majority of harmful substances in CS to be absent in heated tobacco. At the same time, those are presented in substantially smaller concentrations [21, 22].

Conventional cigarette smoke affects the marginal integrity of polymeric tooth restorations and denture bases, such as heat-cured, flexible, titanium-reinforced, and 3D-printed resins, and it’s natural to assume that other effects, like discoloration, surface roughness, and bacterial colonization, might also be affected [23].

Therefore, the possibility of in vitro simulation of the staining susceptibility to smoke could be of interest. Unfortunately, there is a lack of standardization for smoke staining protocols [17]. This study explores the claims that non-heated tobacco could be less harmful and have fewer adverse effects than cigarette smoke. Smokers are more likely to quit when they’re made aware of the adverse effects of smoking than when other strategies are employed to induce the same behaviour [24], according to research on smoking cessation, which was what prompted the authors to perform this study.

In this research the materials used are heat cured acrylic resin and several modifications of it. Conventional heat cured acrylic resin is known for its brittleness and low impact strength. Thus attempts to modify these properties involve the use of metal wires or plates, fibers, particles or metal powder. It was noted that the addition of metal fillers, provides improved strength, thermal conductivity and makes the acrylic resin radiopaque [25]. Thus the addition of titanium nanoparticles to acrylic resin in this study. Flexible acrylic resin, on the other hand, shows lower surface roughness, hardness and impact strength, compared to conventional heat cured acrylic resin [26]. A recent study compared the difference in flexural strength between conventional and 3D printed acrylic resin, finding the latter inferior to the formal [27].

This study compared the effect of CS versus heated tobacco using a custom-made chamber device on the discoloration, surface roughness, and bacterial colonization of different oral prosthesis materials.

**The null hypothesis** was that conventional smoke and heated tobacco exposure would not significantly change the surface roughness, bacterial accumulation, and color change of the study samples and that there is no difference in the effect of both types of smoking.

## Materials and methods

The Research and Ethics Committee of the Faculty of Dentistry, The British University in Egypt, reviewed and approved this research project protocol with project approval number 24 − 005. The sample size was calculated by G*Power software for Windows version 3.1.9.4 based on a previous study [28] The minimum sample size was calculated to be 8 samples per group; it was increased to 10 samples per group to compensate for any defects. The primary outcomes are measuring changes in surface roughness, bacterial accumulation, and dental materials’ color stability due to different smoking types.

### Samples preparation

Four different denture base materials were used to construct one hundred and twenty disc-shaped samples of 1 cm diameter and 2 mm thickness: conventional heat-cured acrylic resin (CA) (Acrostone, Egypt), flexible acrylic resin (FA) (Valplast, Valplast International Corp, USA), heat-cured acrylic resin reinforced with titanium nanoparticles (TA) (TA nanoparticles ( Nanogate, Egypt), and 3D printed acrylic resin (PA) (Nexdent, The Netherlands), composition of materials are shown in (Table 1). Another sixty samples of artificial teeth were used: conventional ready-made acrylic resin teeth (Acrostone, Egypt) and 3D-printed acrylic resin teeth (Nexdent, The Netherlands).

The heat-cured acrylic resin groups were constructed using the conventional compression-molding technique with a long curing cycle (74 °C for 8 h followed by 100 °C for 1 h). For the printed groups (PA and 3D printed teeth), CAD software (Exocad, Darmstadt, Germany) was used to design the samples. Then, the printing angle was set at 90 degrees, and the 3D printer (Anycubic, China) was filled with liquid resin (pink for denture base samples and white teeth samples), and the samples were subsequently printed. The denture base samples were used to assess surface roughness and biofilm formation, while the artificial teeth samples were used to determine color change.

All groups were divided according to the smoking method into three subgroups: the control group with no smoking exposure (I), the conventional smoking exposure group (II), and the heated tobacco exposure group (III).

All samples were stored in artificial saliva at 37 °C for 24 h to simulate the conditions of the oral cavity before any interference. Artificial saliva was obtained by dissolving the following ingredients in one liter of deionized water: Xanthan gum (0.92), KCl (1.2), NaCl (0.85), MgCl2 (0.05), NaH2PO4 (0.13), C8H8O3 (0.13) and CaCl2 (0.13) [29–31].

**Table 1:** Composition of sample materials used

Samples	Conventional heat-cured acrylic resin denture base	Flexible acrylic resin denture base	Titanium-reinforced acrylic resin denture base	3D Printed Acrylic resin denture base	Conventional acrylic resin teeth	3D Printed teeth
Main ingredient	PMMA resin	Nylon based	PMMA resin + Titanium nanoparticles	Dimathacrylate based resin	PMMA	Dimethacrylate based resin
Commercial product	Acrostone	Valplast	Acrostone	NextDent	Acrostone	NextDent

### Baseline measurements

The surface roughness of all denture base samples was measured using a profilometer (JITAI8101 Surface Roughness Tester—Beijing Jitai Tech Detection Device Co. Ltd, China) at cut off 0.25 mm, number of cuts 1and range ± 40 μm. In compliance with ISO 11,562 recommendations for standardization, each sample was measured three times at different locations (the middle and sides), and the average was obtained to get the mean surface roughness values (Ra).

According to the CIE L*a*b* color order system, the three color parameters of each artificial tooth specimen were measured using a VITA Easyshade spectrophotometer Advance 4.01 (VITA Zahnfabric, Bad Sackingen, Germany) at 3 different areas. Mean measurements were then calculated.

### Smoking standardizing device

**Fig. 1:**
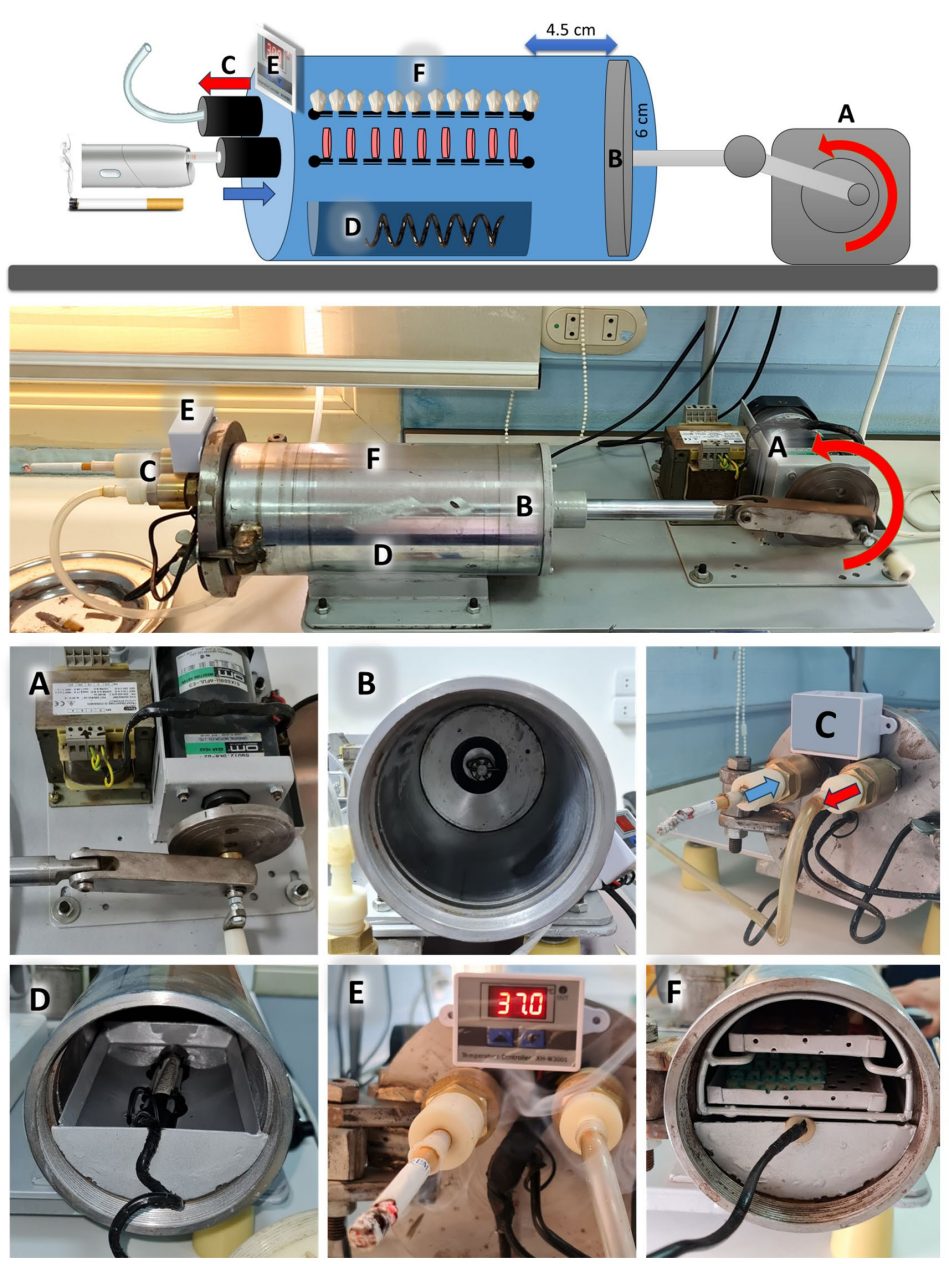
Smoking standardizing device: A motor and gearbox with a crankshaft (**A**), a piston (**B**), two valves that allow inhalation and exhalation in one direction only (**C**), a pool of water with a heater (**D**) connected to a thermal sensor (**E**), and two perforated trays (**F**)

The smoking standardizing apparatus, designed and constructed at The Dentistry Research Center, Faculty of Dentistry, The British University in Egypt, was a crucial tool in this study. It was created to simulate the smoking process to investigate the effects of smoking on different dental materials. The apparatus includes a motor with a gearbox to lower its speed to 2 Hz (2 cycles per second), a crankshaft, and a connecting rod attached to a slider to convert the rotational movement into a 4.5 cm-long linear movement. A stainless-steel cylinder with an internal diameter of 12 cm (6 cm radius) with a piston to generate suction power with about 500 ml volume, simulating the tidal volume taken during smoking [32] was designed. A cigarette or electronic smoking device is attached to a valve that allows inhalation of the smoke in one direction only, simulating the mouth. Another valve allows the exhalation in one direction only, simulating the nose. To simulate the oral cavity, a pool of water with a heater linked to a thermal sensor regulates the temperature between 36.5 and 37.5 °C with 100% humidity [33]. The samples were mounted on 2 perforated trays to allow total exposure of all samples to the smoke equally (Fig. 1).

### Exposure of specimens to smoking

Conventional cigarettes (LM, Philip Morris International Inc., Egypt) and heated tobacco electronic cigarettes (Heets, Russet selection, Philip Morris International Inc., Italy) were used. The samples were exposed to cigarette smoke of 600 cigarettes/heets, representing 30 days of medium smoker behavior (20 cigarettes per day) [34]. Then, the samples were gently washed with distilled water for 1 min.

The control groups were mounted on the perforated trays and were placed in the smoking apparatus. A complete cycle was then performed without smoking.

### Postexposure measurements

The surface roughness of the denture base samples was performed using the same previous parameters. The color parameters of each artificial tooth sample were measured using the same previous method, and then the color change was calculated according to the following formula:

$$\:\Delta E_{2-1}\:=\:\left(\right[\Delta L]^2\:+\:[\Delta a]^2\:+\:[\Delta b\left]^2\right)^{1/2}$$

### SEM assessment

One sample from each group was examined by scanning electron microscopy (Thermo Fisher (USA) Quattro S Felid Emission Gun, Environmental SEM “FEG ESEM”) at the Nanotechnology Research Center at The British University in Egypt to evaluate the surface topography.

### Assessment of bacterial biofilm formation on dental discs by streptococcus mutans strain (S. mutans)

#### Bacterial inoculum preparation

A pure single colony of the reference strain S. (AT ATCC 25175) was used to inoculate 5 ml aliquots in test tubes of brain heart infusion broth supplemented with 2% sucrose. The bacterial cultures were placed in an incubator (Model B 28, BINDER GmbH) at 37 °C for 48 h.

The bacterial culture was then adjusted to an optical density (OD) of 0.09 at 600 nm using brain heart infusion broth in the same medium containing 2% sucrose. The concentration of bacteria was determined using a spectrophotometer (Unicam, UK). The denture base samples were then sterilized and inserted separately into 50 ml falcon tubes. Aliquots of 2 ml of adjusted bacterial suspension were pipetted in these falcon tubes for biofilm formation. Then, the discs containing bacterial suspension were incubated for 48 h at 37 °C [35].

**Fig. 2:**
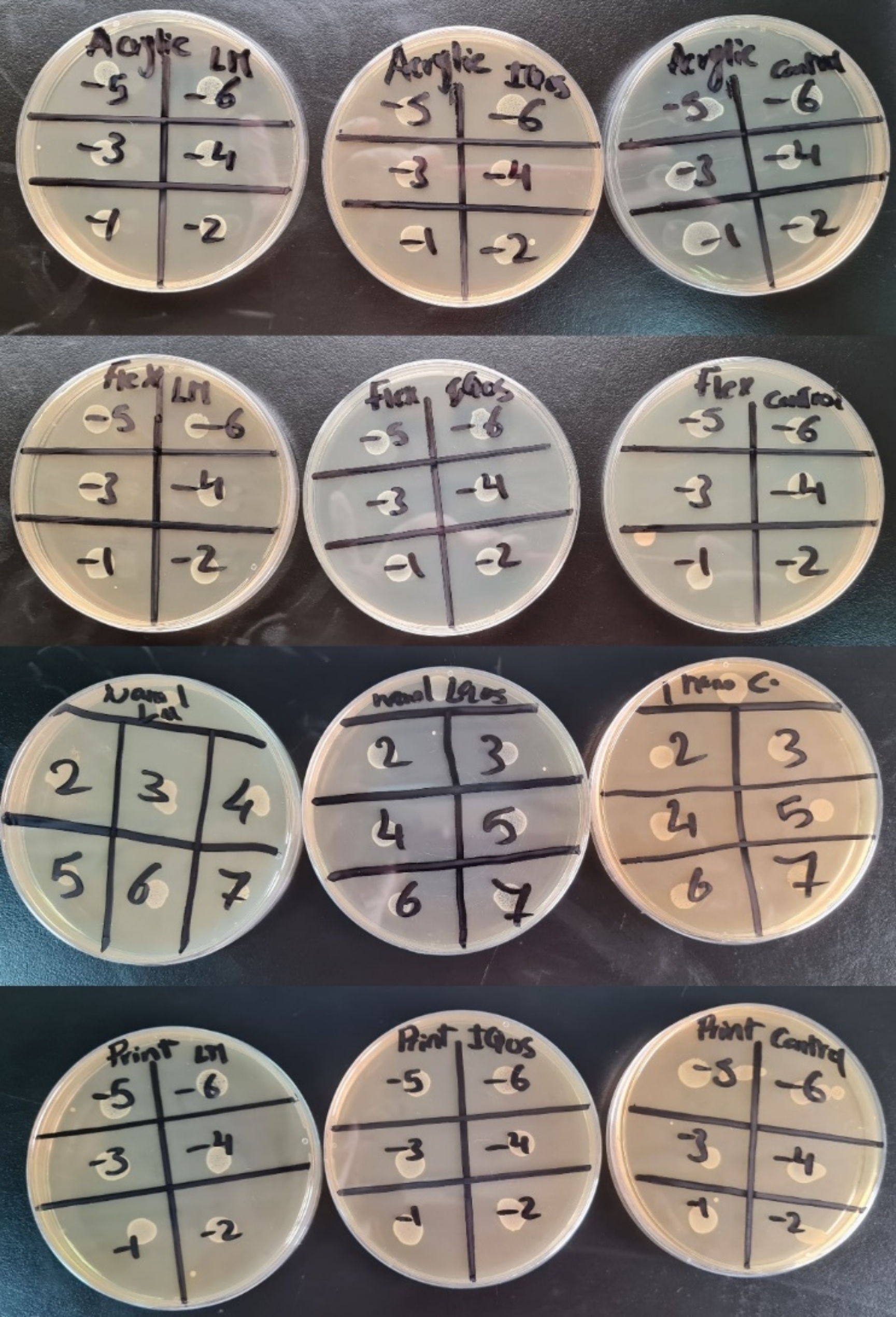
Bacterial biofilm formation of different representative samples with serial dilution on brain heart infusion agar

After that, the samples were aseptically removed from the cultures using sterile forceps and washed gently three times with 0.9% saline to remove the non-adherent bacteria, then transferred to new falcon tubes containing 5 ml of 0.9% saline. To determine biofilm formation attached to the surface of the samples, the falcon tubes were vortexed with a sonicator (Acculab, USA) at 30 g for 3 min to detach microorganisms from the discs. Then, aliquots of 100 µL of the biofilm suspension were serially diluted up to 10^6^. Dilution was performed in triplicates. After that, 10 µL of each diluted suspension was inoculated on brain heart agar plates and incubated at 37 °C for 48 h (Fig. 2). After the incubation, the colony-forming units (CFU) in plates with 30 to 300 typical colonies of S. mutans were counted and then reported in CFU/ml [36].

### Statistical analysis

Statistical analysis of the obtained data was performed using SPSS for Windows (version 26.0; SPSS Inc., Chicago, IL, USA). Paired sample t-test was conducted to determine the change in surface roughness. An independent sample t-test was used to compare color changes between different artificial teeth materials. One-way ANOVA and Tukey post hoc tests were used to determine the effect of various materials and smoking types on surface roughness and bacterial biofilm formation.

## Results

**Fig. 3:**
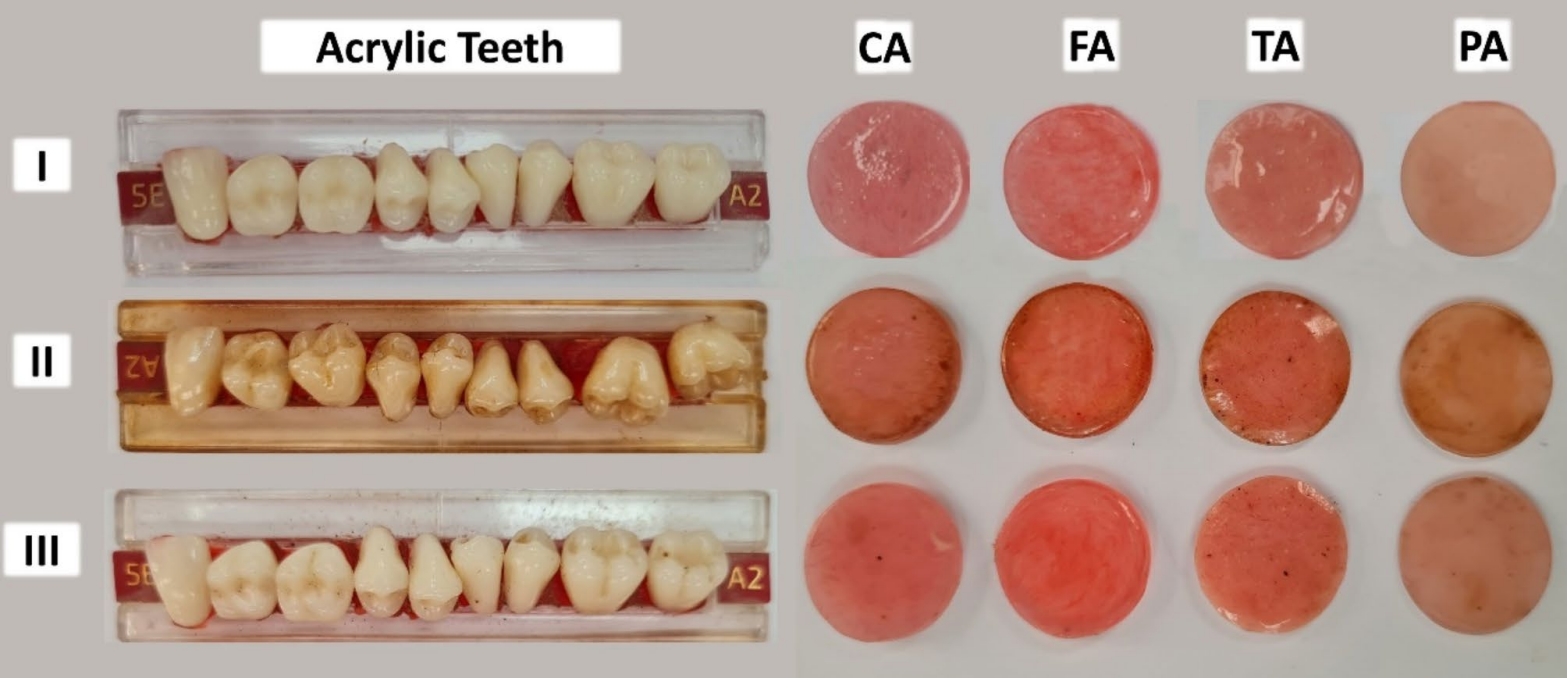
Samples after performing the exposure procedure

Figure 3 shows the samples after performing different exposure procedures: I: the control group with no smoking exposure, II: conventional cigarette smoking exposure, and III: heated tobacco exposure.

### Surface roughness results

The control groups did not show a significant increase in surface roughness for all four types of used denture base materials. However, both types of smoking caused a statistically significant increase in surface roughness. The mean surface roughness values before and after exposure are shown in (Table 2).

**Table 2:** The mean values of before and after surface roughness

		Before exposure	After exposure	*P*-value*
Heat-cured acrylic resin (CA)	Control (I)	0.6556 ± 0.13	0.6564 ± 0.13	0.076
	Conventional cigarette (II)	0.5686 ± 0.17	0.8256 ± 0.32	0.026
	Heated Tobacco (III)	0.7423 ± 0.19	0.9788 ± 0.23	0.012
Flexible resin group (FA)	Control (I)	0.7531 ± 0.19	0.7533 ± 0.19	0.907
	Conventional cigarette (II)	0.7048 ± 0.29	1.061 ± 0.29	0.04
	Heated Tobacco (III)	0.5051 ± 0.23	0.7284 ± 0.26	< 0.01
Titanium-reinforced group (TA)	Control (I)	0.8253 ± 0.2	0.8270 ± 0.2	0.056
	Conventional cigarette (II)	0.5542 ± 0.11	0.7927 ± 0.27	0.024
	Heated Tobacco (III)	0.8578 ± 0.05	0.9668 ± 0.2	0.004
3D-printed group (PA)	Control (I)	0.7422 ± 0.12	0.7435 ± 0.12	0.088
	Conventional cigarette (II)	0.6205 ± 0.13	0.8922 ± 0.33	0.044
	Heated Tobacco (III)	0.864 ± 0.12	0.9842 ± 0.12	0.002

* Significance level set at *p* < 0.05

**Fig. 4:**
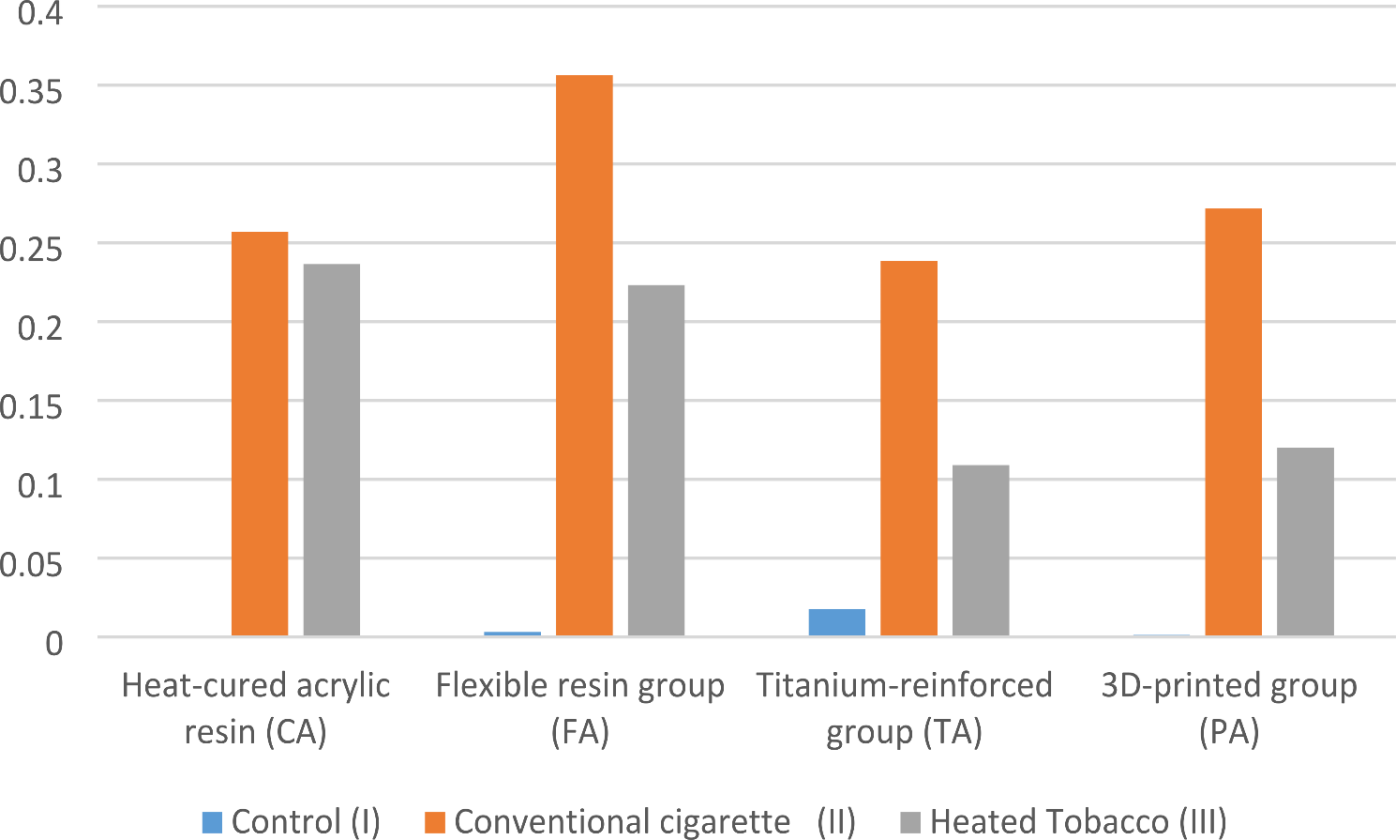
Change in surface roughness (Δ Ra) according to type of smoking

Regarding the effect of the type of smoking on change in surface roughness (Δ Ra) of different denture base materials, there was a statistically significant difference between the control and the conventional cigarette smoking subgroups. However, there was no statistically significant difference between the control and the heated tobacco groups. (Table 3; Fig. 4).

**Fig. 5:**
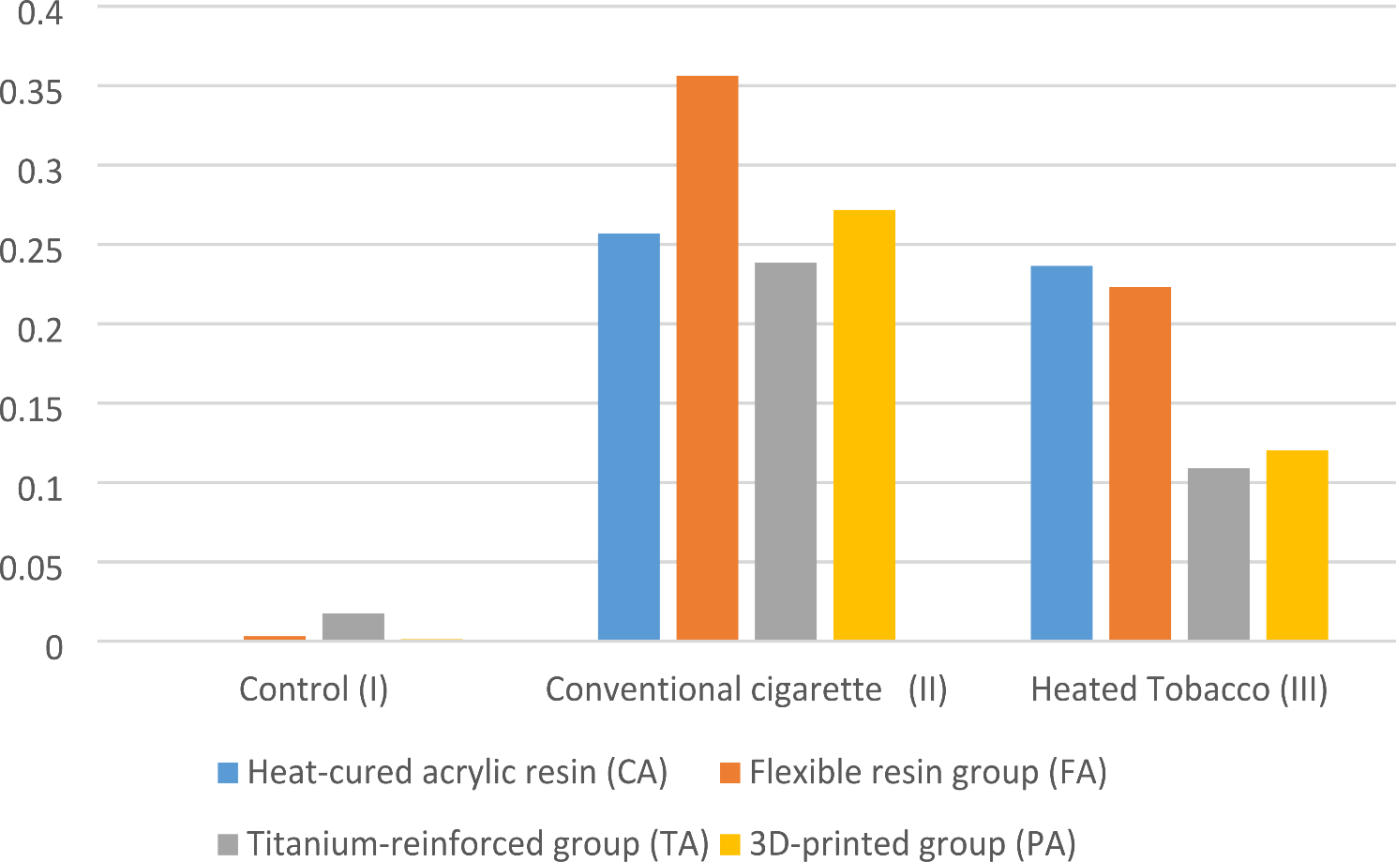
Change in surface roughness (Δ Ra) according to material

Concerning different materials, there was no statistically significant difference between the mean values of Δ Ra of different materials in the control, conventional cigarette smoking, or heated tobacco groups (Table 3; Fig. 5).

**Table 3:** Mean & standard deviation of surface roughness differences (Δ Ra) in µm

	Control (I)	Conventional cigarette (II)	Heated Tobacco (III)	*P*-value*
Heat-cured acrylic resin (CA)	0.0008 ± 0.00121^a^	0.257 ± 0.3063^b^	0.2365 ± 0.2372^ab^	0.03
Flexible resin group (FA)	0.0032 ± 0.0118^a^	0.3563 ± 0.4706^b^	0.2232 ± 0.1266^ab^	0.03
Titanium-reinforced group (TA)	0.0177 ± 0.0025^a^	0.2385 ± 0.2785^b^	0.109 ± 0.0892^ab^	0.015
3D-printed group (PA)	0.0013 ± 0.0021^a^	0.2717 ± 0.3682^b^	0.1202 ± 0.0902^ab^	0.034
P-value	0.15	0.89	0.46	

* Significance level set at *p* < 0.05

** Different superscript in rows indicates statistically significant differences

**Fig. 6:**
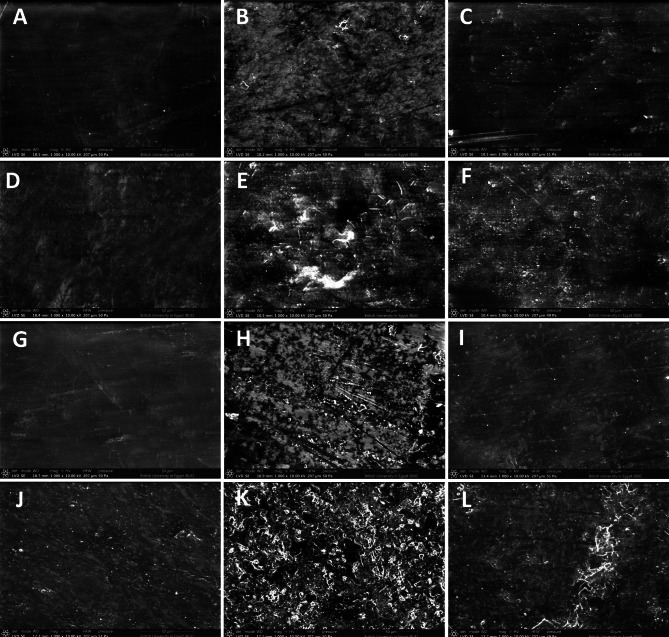
SEM of heat-cured acrylic resin (**A**: control, **B**: CS and **C**: HT), flexible acrylic resin (**D**: control, **E**: CS and **F**: HT), heat-cured acrylic resin reinforced with titanium nanoparticles (**G**: control, **H**: CS and **I**: HT), and 3D printed acrylic resin (**J**: control, **K**: CS and **L**: HT)

The surface topography images of the studied samples at 1000X are presented in (Fig. 6). The CS groups showed significant change in the surface topography with increased pitting of the surface compared to the control groups. The change in the topography of the surface of the samples was almost identical for all types of denture base materials. Also, the HT groups presented increased pitting than the control groups but to a lesser amount than that of the CS groups.

### Bacterial accumulation test

**Fig. 7:**
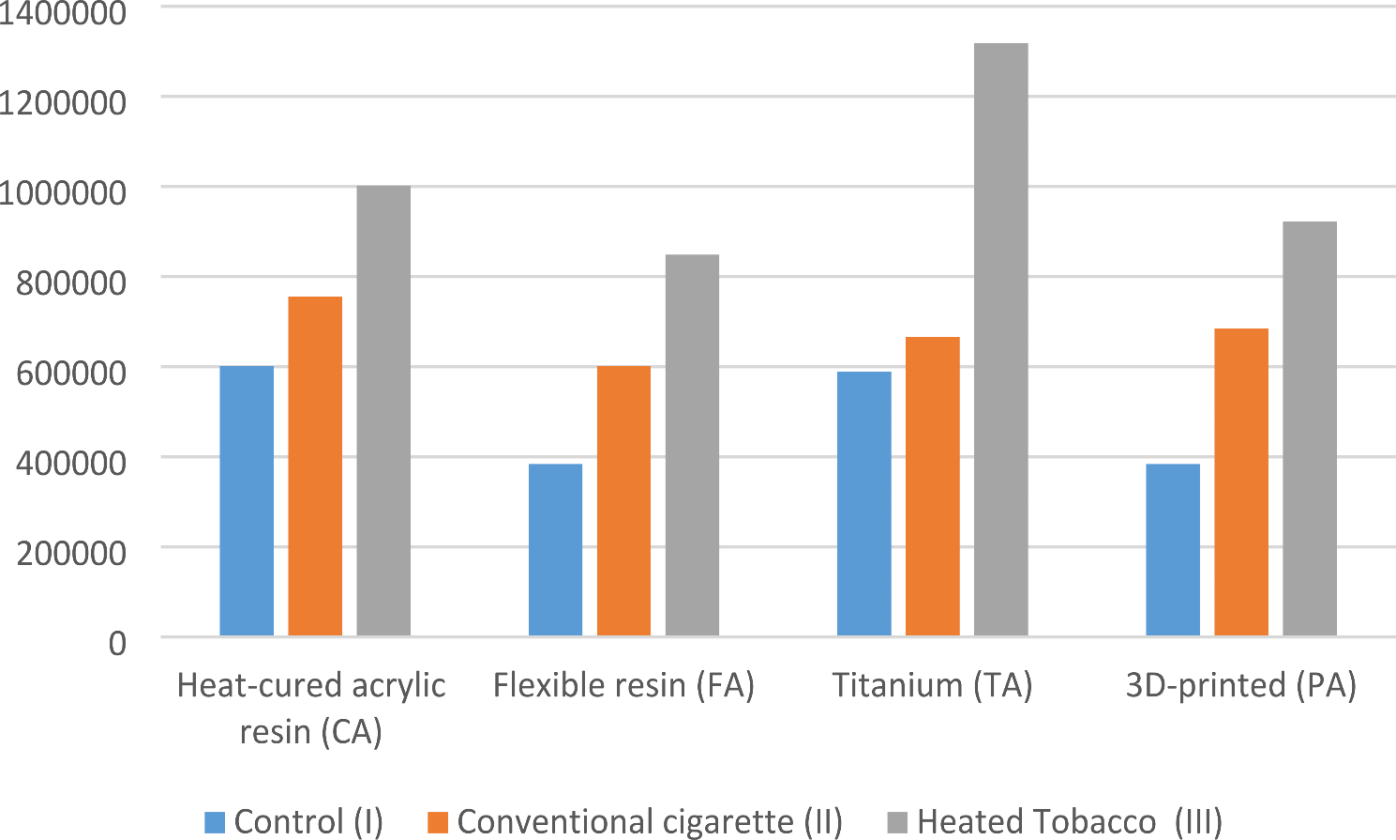
Bacterial accumulation according to type of smoking

Using ANOVA and Tukey as post-hoc tests, it was found that there was a statistically significant difference between all smoking subgroups. In the CA, FA, and PA groups, the heated tobacco subgroup (CA III) showed the highest level of bacterial accumulation, while the control groups showed the least. For the TA group, the heated tobacco subgroup showed the significantly highest level of bacterial accumulation, and there was no difference between the control and the conventional cigarette smoking groups (Table 4; Fig. 7).

In the control subgroup (I), there was a statistically significant difference between all groups. The (FA I) and the (PA I) subgroups showed significantly higher bacterial accumulation than the (CA I) and the (TA I) groups.

In the conventional cigarette smoking subgroup (II), there was a statistically significant difference between all groups, with the (CA II) showing the highest significant bacterial accumulation, followed by the (TA II) and (PA II) and the (FA II) showing the least significant.

**Fig. 8:**
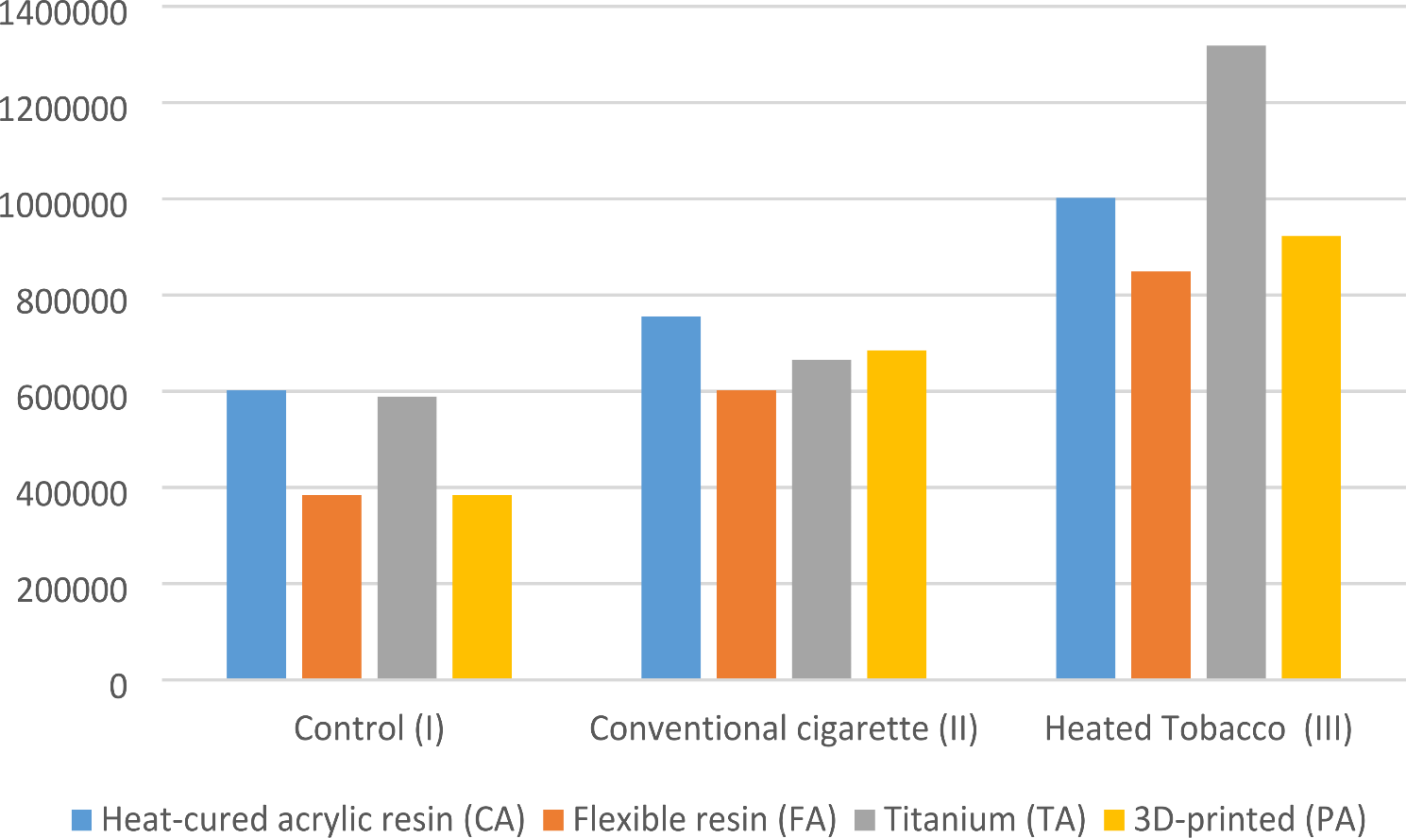
Bacterial accumulation according to material

For the heated tobacco subgroup (III), there was a statistically significant difference between all subgroups. The (TA III) showed the highest significant bacterial accumulation, and the (FA III) showed the significantly least bacterial accumulation. There was no statistically significant difference between the (CA III) and (PA III) or the (PA III) and (FA III) (Table 4; Fig. 8).

**Table 4:** Mean & standard deviation of bacterial accumulation in (CFU/ml)

	Control (I)	Conventional cigarette (II)	Heated Tobacco (III)	*P*-value*
Heat-cured acrylic resin (CA)	601,600 ± 15,878^a1^	755,200 ± 14,046^b1^	1,002,000 ± 24,466^c1^	< 0.001
Flexible resin (FA)	384,000 ± 10,486^a2^	601,600 ± 16,473^b2^	848,800 ± 22,608^c2^	< 0.001
Titanium (TA)	588,800 ± 23,791^a1^	665,600 ± 27,181^a3^	1,318,400 ± 247,367^b3^	< 0.001
3D-printed (PA)	384,000 ± 19,917^a2^	684,800 ± 29,744^b3^	922,800 ± 34,990^c12^	< 0.001
P-value*	< 0.001	< 0.001	< 0.001	

* Significance level set at *p* < 0.05

** Different superscript in rows indicates statistically significant differences between types of smoking

*** Different numbers in columns indicate statistically significant differences between different materials

### Color change

**Fig. 9:**
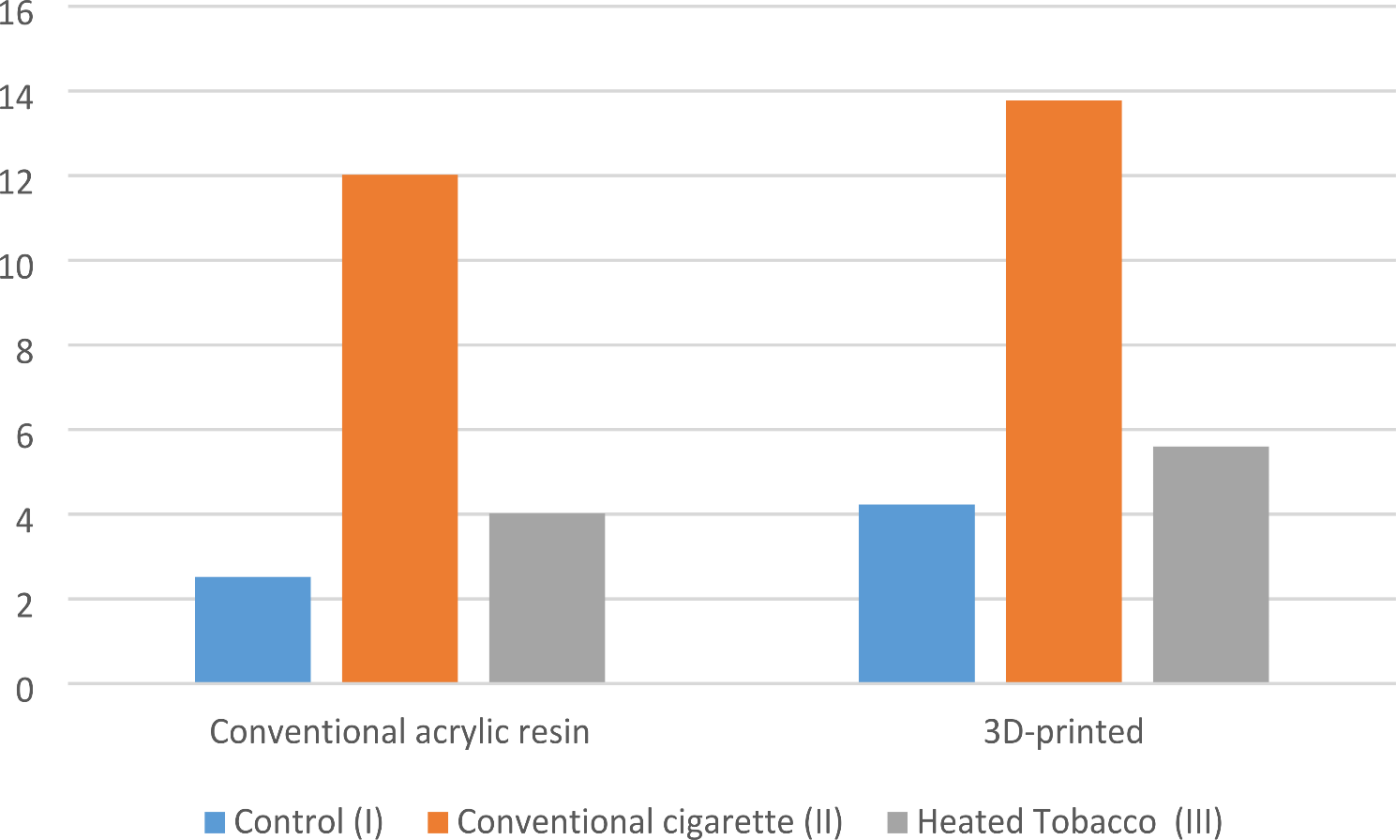
Color change according to type of smoking (Δ E)

For both types of teeth, the conventional cigarette smoking groups showed statistically higher significant differences in the mean values of color change (ΔE) than the control and the heated tobacco groups (Table 5; Fig. 9).

**Fig. 10:**
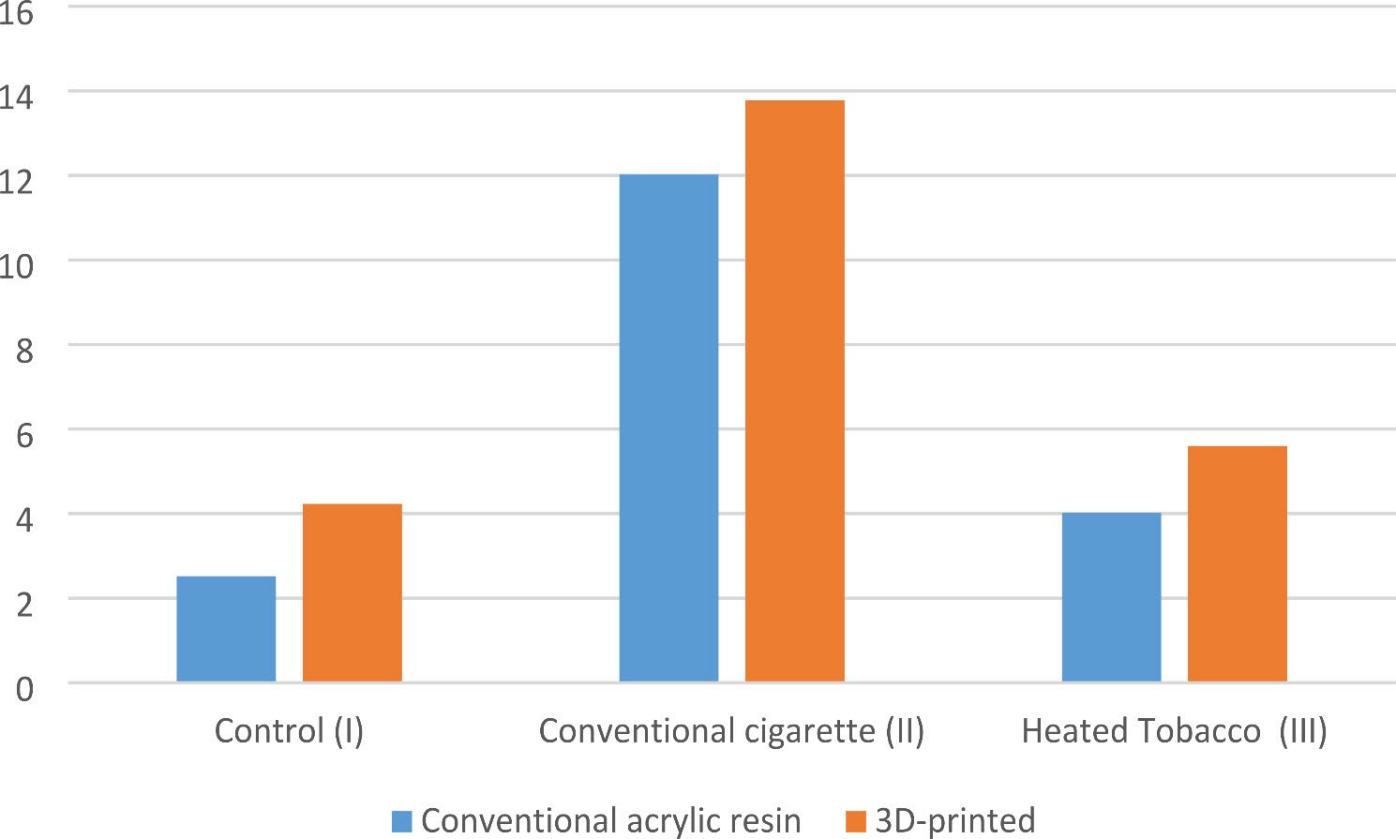
Color change according to material (Δ E)

Concerning the type of smoking groups, there was no statistically significant difference between the conventional acrylic resin and the 3D-printed groups (Table 5; Fig. 10).

**Table 5:** Mean & standard deviation of color change

	Control subgroup (I)	Smoking cigarette Subgroup (II)	Heated Tobacco subgroup (III)	*P*-Value
Conventional acrylic resin	2.52 ± 1.18^a^	12.02 ± 2.48^b^	4.02 ± 2.78^a^	< 0.001
3D-printed	4.23 ± 2.28^a^	13.77 ± 3.55^b^	5.6 ± 2.42^a^	< 0.001
P value	0.0508	0.2182	0.1948	

* Significance level set at *p* < 0.05

** Different superscript indicates statistically significant differences

## Discussion

An in vitro study design was employed to control all the factors and enable accurate data collection. The study evaluated and compared the effect of conventional cigarette smoking and heated tobacco on the surface roughness, bacterial accumulation, and color stability of different denture bases and teeth materials.

The null hypothesis of the study were rejected as significant differences were found among different groups in surface roughness, biofilm formation and color change.

The results showed that conventional cigarette smoking and heated tobacco significantly increased the surface roughness of different denture base materials. Although conventional smoke increased the surface roughness by a greater degree, this difference was not statistically significant. These results are consistent with previous studies, which state that smoking of all types affects the surface roughness of dental materials and that tobacco consumption of all types is associated with tooth discoloration and changes in the surface properties of dental materials [28, 37]. This finding was supported by the SEM images, which showed that all CS groups had a noticeable increase in the pitting of the acrylic surface.

With CS, these changes were attributed to the deposition of cigarette substances on the surface of the acrylic resin [38]. When burning the cigarette, the resultant smoke contains multiple components, such as carbon monoxide, carbon dioxide, nicotine, ammonia, nickel, arsenic, tar, and heavy metals such as lead and cadmium [1, 39, 40]. Another possible explanation may be due to the increase in temperature within the smoking chamber, i.e., the thermal effects of smoking, as reported in a previous study [10]. According to Mathias P et al., the tar of cigarettes contains aromatic hydrocarbons that have a surface-dissolving action on the polymeric materials. Polymeric materials are insoluble in oral fluids but are soluble to some extent in aromatic hydrocarbons [12]. From another point of view, there is a possibility that cigarette smoke will get mixed with saliva, which may produce an acidic pH solution, damaging the surface integrity of the materials [2]. Previous studies have claimed that heated tobacco is a significantly safer smoking option in terms of product release due to the absence of tar, which was identified as a leading cause of increased surface roughness and material discoloration [1, 11, 12, 41, 42]. However, in our study, although the increase in surface roughness after exposure to HT was less than that after CS, this difference was insignificant.

This study also showed a significant increase in bacterial biofilm formation on all denture base materials CS and HT, which could be related to surface roughness. The clinical threshold value of surface roughness (Ra) for plaque retention on intraoral materials was 0.2 μm as advocated by Bollen C et al., [43]. In accordance, below this value, no further reduction in plaque accumulation was expected, but above it, a proportional increase occurred [44]. Other studies have previously stated that surface irregularities provide an environment that promotes bacterial colonization and biofilm formation [45–47]. Surface roughness increases surface area, hydrophobicity, and surface energy, which, in turn, affects the mechanism of the bacteria’s attachment to that surface and its adhesion [48, 49]. The increase in bacterial biofilm formation was more significant in all HT groups than in the CS groups. It was previously claimed by another study that e-smoke promoted the growth of S. mutans, the expression of virulent genes, and the adhesion to and formation of biofilms on tooth surfaces, supporting the increase in bacterial biofilm formation [50, 51].

The increase in surface roughness has long been proven to cause resins to have a rougher surface and a resultant color change. We can see this when we compare resins to dental ceramics, whose highly glazed and polished surfaces result in greater color stability. In comparison, resins are more porous and have a less polished outer surface [40]. A recent study found that 3D printed resins showed inferior mechanical properties and higher water solubility than conventional heat cured acrylic resin, even before external stimuli, which might cause us to expect a significant difference between the two materials when exposed to smoking, however this was not the case with our study, where the difference between the 2 materials was statistically insignificant [27].

Spectrophotometers often report color using the CIELAB color system, representing the international standard for color measurement. It is currently one of the most popular and widely used color spaces. It is well suited for determining minor color differences [52]. ΔE values less than 1 were regarded as undetectable by the human eye. Color differences of 3.3 > ΔE > 1 may be detectable by a skilled operator but were considered clinically acceptable. On the other hand, values of ΔE > 3.3 would be detectable by a nonskilled person and, therefore, considered clinically unacceptable [53, 54]. In this study, all groups except the conventional acrylic resin artificial teeth showed ΔE > 3.3.

Both heated tobacco and CS caused a significant color change in 3D-printed teeth. This coincides with another study whose results found that the most remarkable changes in surface roughness were observed in their 3D-printed samples, followed by the heat-polymerized samples, and that these changes can alter their translucency and opacity, thus affecting their color. In contrast, Alfouzan et al. studied and compared the color stability of 3D-printed and conventional heat-polymerized acrylic resins in general following aging, mechanical brushing, and immersion in a staining medium, and found that color changes of 3D-printed denture resins were low compared to conventional heat polymerized resin [55]. Flexible resin, on the other hand, was found by another study to be the least staining denture base material as compared to conventional heat cured acrylic resin [56].

The CS caused a significant color change in conventional acrylic resin and 3D-printed artificial teeth compared to the heated tobacco groups, which could be attributed to the latter’s absence of tar. Several studies report that cigarette smoking affects the color of natural teeth and dental materials, including denture teeth [13, 18]. The results of this study were consistent with those of Mathias P et al., [10] Wasilewski MA, et al., [57] and Mathias P, et al., [12]. These studies evaluated the effect of tobacco smoke on the color of composites. A slight color change occurred in all control group samples, which was relative to baseline; this was assumed to be due to the thermal effect of the immersion temperature and due to water absorption [58] and due to mucin, which is one of the components of the artificial saliva [11]. According to Craig [59], polymeric teeth are insoluble in oral fluids, but they are soluble to some extent in aromatic hydrocarbons. According to Mathias P, et al., [12] tar contains aromatic hydrocarbons. So, it was deduced from this study that such surface-dissolving substances might be causative factors of discoloration. Also, there was a possibility that the cigarette smoke mixed with saliva may have produced an acidic pH solution, which might have damaged the surface integrity of the materials, thus creating favorable conditions for discoloration [2].

## Conclusion

Despite this study’s limitations, we have concluded that conventional cigarette smoking and heated tobacco affect the surface roughness, bacterial biofilm formation, and color changes of dental materials.

## Data Availability

No datasets were generated or analysed during the current study.
